# Use of thiazide diuretics for the prevention of recurrent kidney calculi: a systematic review and meta-analysis

**DOI:** 10.1186/s12967-020-02270-7

**Published:** 2020-02-28

**Authors:** Dan-feng Li, Yu-lu Gao, Hong-chao Liu, Xiao-chen Huang, Rui-fang Zhu, Chang-tai Zhu

**Affiliations:** 1 Department of Transfusion Medicine, Shanghai Jiao Tong University Affiliated Sixth People’s Hospital, Shanghai, 200233 China; 2 Department of Laboratory Medicine, Kunshan Hospital Affiliated to Nanjing University of Traditional Chinese Medicine, Kunshan, 215300 Jiangsu China; 3 School of Nursing, Shanxi Medical University, 56 Xinjian South Road, Yingze District, Taiyuan, 030001 Shanxi China

**Keywords:** Thiazide diuretics, Kidney calculi, Randomized controlled trial, Placebo

## Abstract

**Background:**

Thiazide diuretics reduce the risk of recurrent kidney calculi in patients with kidney calculi or hypercalciuria. However, whether thiazide diuretics can definitely prevent recurrent kidney calculi remains unclear. We aimed to evaluate the effect and safety of thiazide diuretics on recurrent kidney calculi.

**Methods:**

The PubMed, Cochrane Library, and EMBASE databases were systematically searched using the keywords *thiazide diuretics* and *kidney calculi* to identify randomized controlled trials (RCTs). The primary outcome was the incidence of recurrent kidney calculi, and the secondary outcome was the 24-h urinary calcium level. The pooled risk ratio (RR), risk difference (RD), standardized mean difference (SMD), and 95% confidence interval (CI) were calculated. The evidence quality was graded using the GRADE criteria, and recommendations for recurrent kidney calculus prevention using thiazide diuretics were reassessed.

**Results:**

Eight RCTs involving 571 patients were included. The pooled RR for the incidence of kidney calculi in the thiazide diuretic groups was 0.44 (95% CI 0.33–0.58, *P *< 0.0001) compared to that in the placebo and untreated groups; the pooled RD was − 0.23 (95% CI − 0.30 to − 0.16, *P *< 0.0001). The pooled SMD for the 24-h urinary calcium level was − 18.59 (95% CI − 25.11 to − 12.08, *P *< 0.0001). The thiazide diuretic groups had a high incidence of adverse reactions and low tolerance. The evidence quality for decrease in kidney calculus incidence using thiazide diuretics was low, while that for the 24-h urinary calcium level decrease among those with recurrent kidney calculi was moderate, and that for the decrease in kidney calculus incidence using short-acting and long-acting thiazide diuretics was low. The overall strength of recommendation for prevention of recurrent renal calculi using thiazide diuretics was not recommended. The subgroup and sensitivity analysis findings were robust.

**Conclusions:**

Long-term use of thiazide diuretics reduces the incidence of recurrent renal calculi and 24-h urinary calcium level. However, the benefits are insufficient, and the evidence quality is low. Considering the adverse effects, poor patient compliance, and economic burden of long-term medication, their use in preventing recurrent kidney calculi is not recommended.

## Background

Kidney calculi are a common urinary system disease, and their incidence is increasing annually [1]. In the clinical guideline of the American College of Physicians, the prevalence of kidney calculi in men and women was 13% and 7%, respectively, and the 5-year recurrence rate of untreated kidney calculi was 35–50% [2]. Correspondingly, the high recurrence rate yielded a certain economic burden to patients with kidney calculi. Kidney calculi are caused by abnormal accumulation of some crystalline substances, such as calcium, oxalic acid, uric acid, and cystine, and organic matrices, such as matrix A and acid mucopolysaccharide, in the kidney. Most kidney calculi are composed of calcium; calcium oxalate stones are the most common, accounting for 74.8% of all cases of stones [3]. In response to increasing incidence of kidney calculi, various interventions have been performed to prevent the occurrence of stones, including dietary interventions and medical treatments [4–6]. Among them, thiazide diuretics are the commonly used drugs for preventing recurrent kidney calculi [7, 8]. However, some clinical trials reported that thiazide diuretics had no significant prophylactic effect compared with the placebo [9, 10]. We also found that the recommended grade of thiazide diuretics to prevent recurrent kidney calculi was not consistent among guidelines [2, 7, 8, 11]. Further, no meta-analyses have yet been conducted to prove that thiazide diuretics can prevent recurrent renal calculi. Therefore, we performed a systematic review and meta-analysis of clinical trials that investigated thiazide diuretics for the prevention of recurrent renal calculi to provide evidence-based medical data for use in clinical practice. We aimed to evaluate the effect and safety of thiazide diuretics on recurrent kidney calculi patients, comparing the incidence of recurrent kidney calculi and the 24-h urinary calcium level with that of placebo or no medication group in randomized controlled trials (RCTs).

## Methods

### Trial search

Two researchers independently searched all studies with keywords of thiazide diuretics and kidney calculi published in the PubMed, EMBASE, and Cochrane Library databases. The following search terms were used: “kidney stones”, “kidney calculi”, “renal calculi”, “nephrolith”, “thiazide diuretics”, “sodium chloride cotransporter inhibitors”. The article search was limited by study design of RCTs. The search was performed until December 31, 2018. In addition, the references of the included studies were also retrieved to supplement the relevant research, including gray literatures (e.g., clinical trials). When the opinions of the two researchers differed, a third researcher was consulted. When there were other languages used, we sought help from linguists.

### Inclusion and exclusion criteria

The inclusion and exclusion criteria for the studies were determined prior to data extraction. The inclusion criteria included the following: (1) RCTs with thiazide diuretic administration as the intervention and placebo or no medication as the control condition; (2) patients with renal calculi or hypercalciuria as the study subjects; and (3) test indicators including at least one of the following: number of patients with new stones, 24-h urinary calcium level, 24-h urinary oxalate level, and serum calcium level. Conversely, the exclusion criteria included the following: (1) non-RCTs; (2) RCTs without a treatment group or placebo group; (3) trials with incomplete or no data available; and (4) other study types, e.g., abstract, case report, and review.

### Risk of bias assessment

Two investigators independently searched for studies using different search strategies and screened for studies that met the inclusion criteria. The researchers evaluated the quality of the included RCTs using the Cochrane Library risk bias assessment tool. The seven items used to assess bias in each trial included random sequence generation, allocation concealment, double blindness of participants and trial performers, blindness of outcome assessment, incomplete outcome data, selective reporting, and other biases. Each quality item was divided and categorized into high risk, undefined risk, and low risk. The quality of the included trials was rated as low quality, high quality, or medium quality according to the following criteria: (1) if random sequence generation or allocation concealment was assessed to be of a high risk, the trial would be considered to be of low quality regardless of the risk of other projects; (2) if random sequence generation and allocation concealment were assessed to be of a low risk, and all other items were assessed to be of a low or an undefined risk, the test would be of high quality; and (3) if the tests did not meet the high risk or low risk criteria, the quality of the trial was considered moderate [12].

### Data extraction

Two investigators extracted the following data from the included studies: primary author; year of publication; sex, age; sample size; interventions; control group; number of lost visits; and follow-up time. When the trials were greater than two sets or had multi-factor designs, we only extracted content relevant to this study.

### Statistical analysis

We performed a meta-analysis and calculated the relative ratio (RR), risk difference (RD), standardized mean difference (SMD), and 95% confidence interval (CI). The pooled RR and RD were used to estimate the efficiency of thiazide diuretics on recurrent kidney calculi by using Mantel–Haenszel method. The pooled SMD were used to evaluate the effect of thiazide diuretics on 24-h urinary calcium level by using inverse variance method. We used *I*^2^ statistics to assess statistical heterogeneity. An *I*^2^ value of 0–25% indicates no significant heterogeneity; 26–50%, low heterogeneity; 51–75%, moderate heterogeneity; and > 75%, high heterogeneity [13]. Data were pooled using the fixed-effects model when the *I*^2^ value was < 50%; data were pooled using the random-effects model when the *I*^2^ value was > 50%. The test level was set at *α *= 0.05; *P* values of 0.05 were considered to indicate that the difference was statistically significant. We used the Review Manager 5.2 software (The Cochrane Collaboration, Oxford, UK) to perform the meta-analysis and forest plot analysis and the Stata 13.0 software (Stata Corp, College Station, TX) to conduct the publication bias test (Egger’s test). Evidence quality grading was performed for each outcome measure with reference to the GRADE criteria, and recommendations for the prevention of recurrent kidney calculi using thiazide diuretics were reassessed on the basis of a decision table formed according to the recommendations of the WHO Handbook for Guideline Development [14]. To assess whether the efficacy of thiazide diuretics in preventing recurrent kidney calculi is related to their clinical features, we performed a subgroup analysis based on the duration of drug action; we also did a subgroup analysis based on quantitative methods of 24-h urinary calcium. For the robustness of the results, we conducted a sensitivity analysis.

## Results

### Study search

**Fig. 1:**
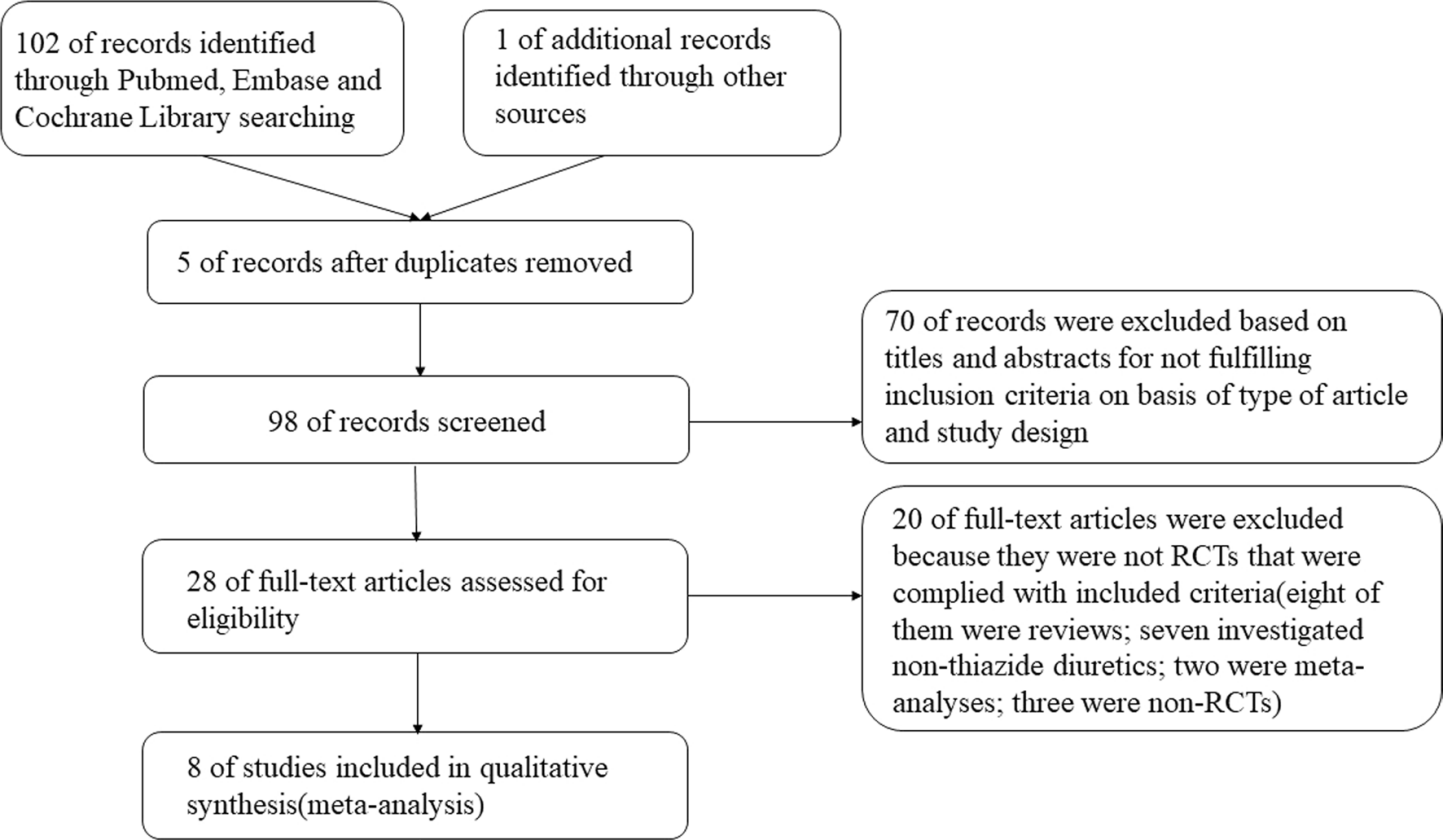
Literature search and screening process

A total of 103 records were searched according to the search strategy (Additional file 1: Table S1), and 28 of them were related to thiazide diuretics for preventing kidney calculi after screening of the titles and abstracts (Fig. 1). Eight of them were reviews; seven investigated non-thiazide diuretics compared with a control condition; two reported failure to reduce the incidence of renal calculi; two were meta-analyses; three were non-RCTs; and six reported reduction of the occurrence of kidney calculi. We finally included eight RCTs conducted on thiazide diuretics [9, 10, 15–20].

### Characteristics and risk of bias

**Fig. 2:**
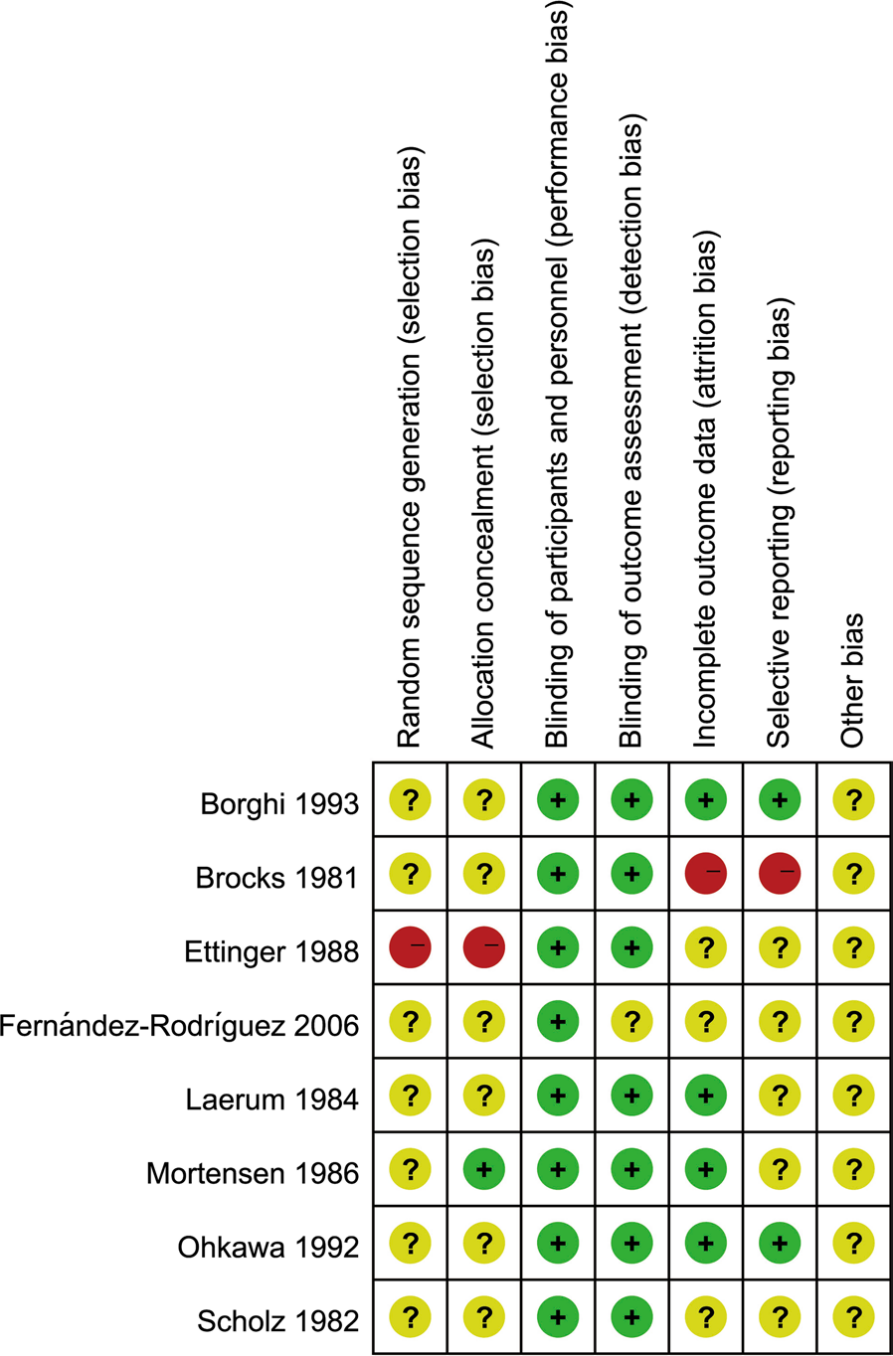
Risk of bias summary for included studies

Among the included articles, there were seven studies published in English language [9, 10, 15–17, 19, 20] and one in Spanish language [18]. Seven of them had patients with recurrent calcium stones as the subjects [9, 10, 16–20]; one trial had patients with idiopathic hypercalciuria as the subjects [15]. Table 1 shows the specific characteristics and data of the studies included. The patients and experimenters were both blinded to the study data. Seven papers did not report the generation of random sequence [9, 10, 15–18, 20] and were of moderate quality. One study used the medical record number to assign patients into groups [19], which was of low quality. The overall quality of the studies was moderate (Fig. 2).

**Table 1:** Characteristics of the included trials and participants

Author	Year	Patients	Gender (M/F)	Mean age (year)	Sample	Events/total (intervention)	Events/total (placebo)	Drugs (intervention)	Drugs (control)	Lost visits	Percent of lost visits	Follow-up (month)
Borghi et al. [20]	1993	Recurrent calcium stones	I: 18/7 C: 20/5	I: 46.5 C: 42.8	40	3/19	9/21	Indapamide	No treatment	10	20%	36
Brocks et al. [10]	1981	Recurrent calcium stones	NR	16–49	62	5/33	5/29	Bendroflumethiazide	Placebo	0	0	48
Ettinger et al. [19]	1988	Recurrent calcium stones	NR	T: L 49.8, H 49.3 C: 48.9	73	6/42	14/31	Chlorthalidone	Placebo	NR	NR	36
Fernández-Rodríguez et al. [18]	2006	Recurrent calcium stones	NR	NR	100	16/50	28/50	Hydrochlorothiazide	No treatment	0	0	36
Laerum et al. [17]	1984	Recurrent calcium stones	38/12	T: 45.8 C: 42.7	48	5/23	12/25	Hydrochlorothiazide + KCl	Placebo	2	4%	12–51
Mortensen et al. [16]	1986	Recurrent kidney stones	All male	20–49	22	0/12	4/10	Bendroflumethiazide + KCl	Placebo	5	18.5%	72
Ohkawa et al. [15]	1992	Calcium stones with hypercalciuria	I: 45/37 C: 52/41	I: 48.7 C: 46.9	175	11/82	41/93	Trichlormethiazide	No treatment	35	16.7%	6–68
Scholz et al. [9]	1982	Recurrent calcium calculi	I: 14/11 C: 17/9	I: 46 C: 41	51	6/25	6/26	Hydrochlorothiazide	Placebo	3	5.6%	12

*I* intervention group, *C* control group, *NR* not reported, *L* low dose group, *H* high dose group, *KCl* potassium chloride

### Incidence of recurrent stone events

**Fig. 3:**
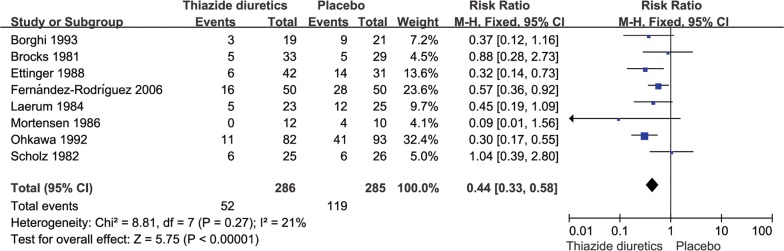
Meta-analysis of the incidence of stones in the thiazide diuretic group versus placebo group

**Fig. 4:**
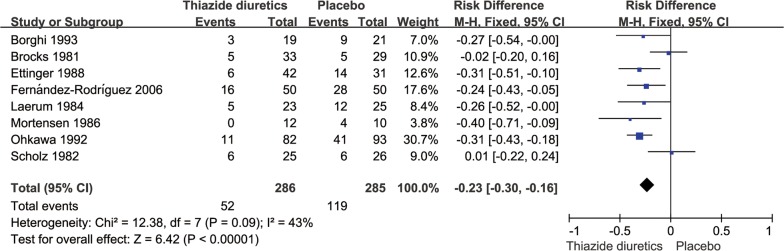
Risk difference forest plot of stone incidence in thiazide diuretic group versus placebo group

There were 286 cases of patients with recurrent kidney calculi in the thiazide diuretic groups and 52 cases of new stones, accounting for 18.2% of all patients; conversely, there were 285 cases in the placebo and untreated groups and 119 cases of new stones, accounting for 41.2% of all patients. The pooled RR for the incidence of renal calculi in the thiazide diuretic groups was 0.44 (95% CI 0.33–0.58, *P *< 0.0001, *I*^2^= 21%; fixed-effects model; Fig. 3); the pooled RD was − 0.23 (95% CI − 0.30 to − 0.16, *P *< 0.0001, *I*^2^= 43%; fixed-effects model; Fig. 4).

### Twenty-four-hour urinary calcium level

**Fig. 5:**
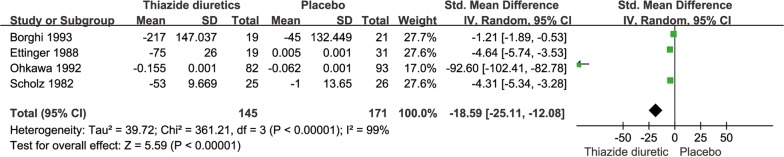
SMD forest plot of 24-h urinary calcium level in thiazide diuretic group versus placebo group

The pooled SMD for the 24-h urinary calcium level was − 18.59 (95% CI − 25.11 to − 12.08, *P *< 0.0001, *I*^2^= 99%; random-effects model; Fig. 5). Furthermore, Mortensen et al. [16] reported that the 24-h urinary calcium level of seven patients in their thiazide diuretic group was reduced by 20–25%; none of their control group patients had a urinary calcium level reduction of > 20%.

### Subgroup analyses

**Fig. 6:**
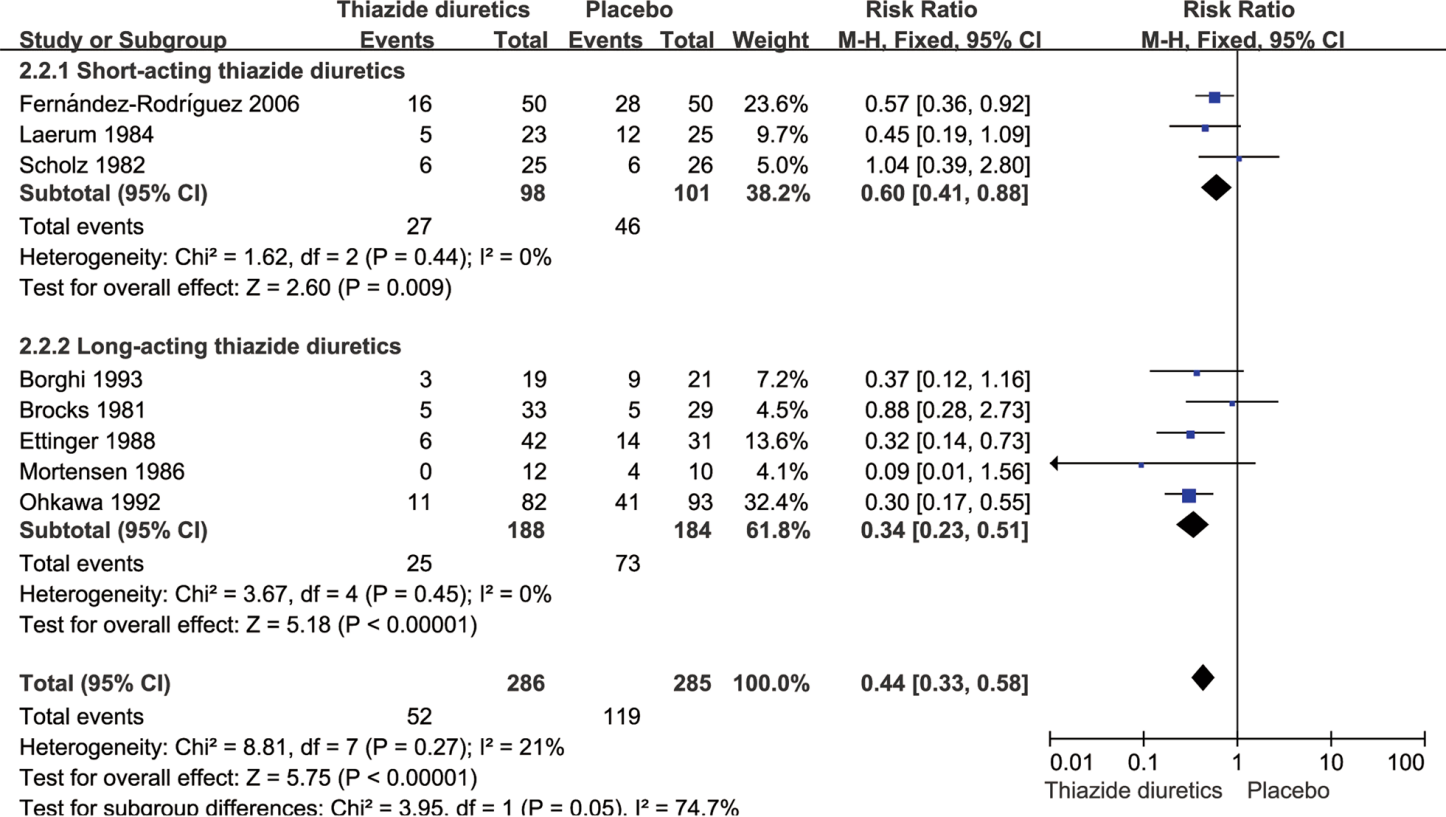
Subgroup analysis of thiazide diuretics based on duration of drug action

**Fig. 7:**
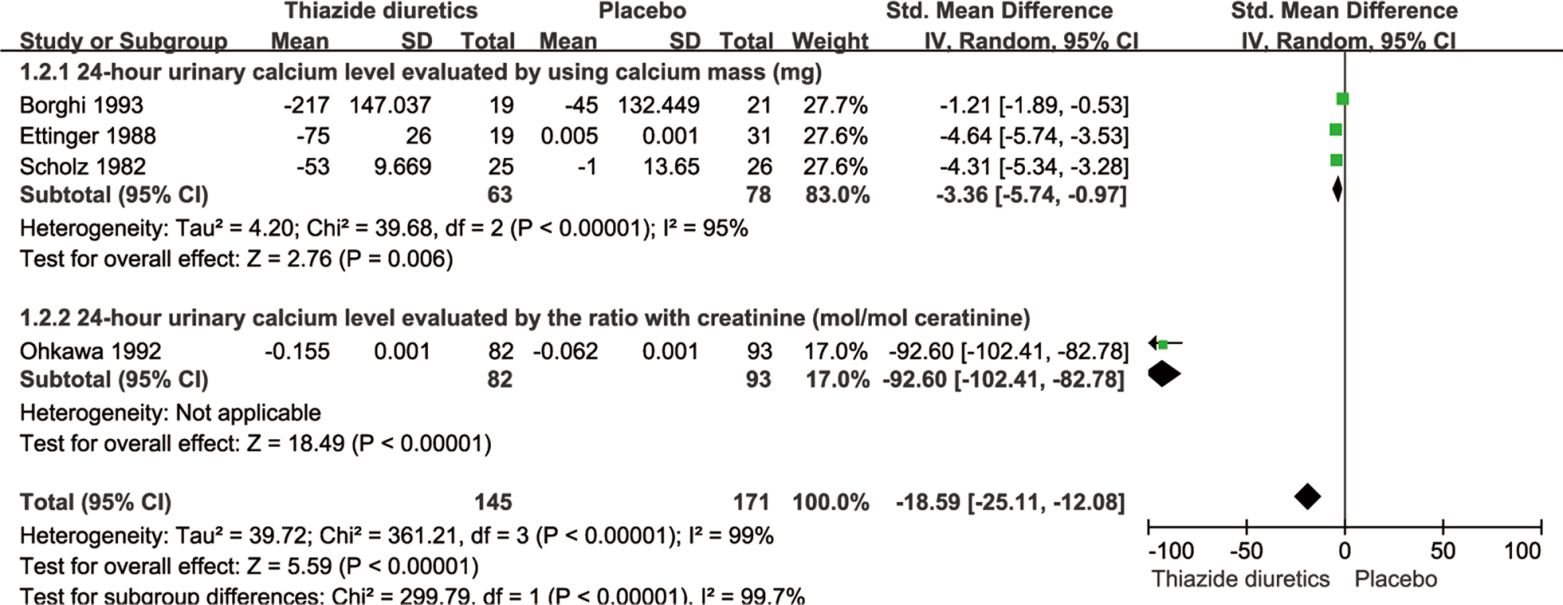
Subgroup analysis of 24-h urinary calcium level

The subgroup analysis showed that there was no significant difference in the incidence of recurrent renal calculi between short-acting [9, 17, 18] and long-acting thiazide diuretics [10, 15, 16, 19, 20] (*P *= 0.05; Fig. 6). Subgroup analysis revealed that there was a significant difference between 24-h urinary calcium tested by using calcium mass (mg) and the ratio with creatinine (mol/mol) (*P *< 0.00001, Fig. 7).

### Sensitivity analysis

**Fig. 8:**
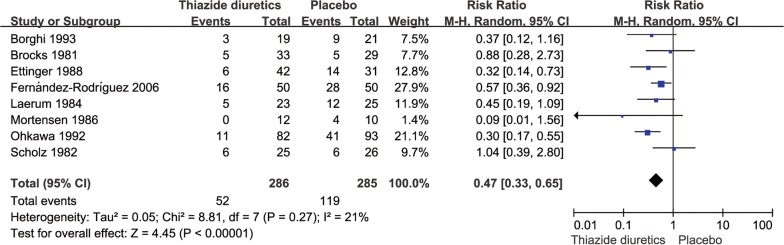
Meta-analysis of the incidence of stones by random-effects model in the thiazide diuretic group versus placebo group

**Fig. 9:**
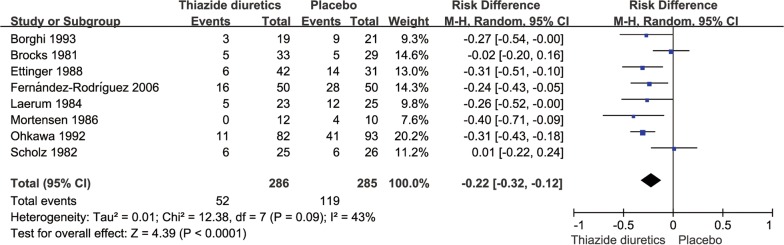
Risk difference forest plot of stone incidence by random-effects model in thiazide diuretic group versus placebo group

The sensitivity analysis showed that the results did not change significantly after changing the fixed-effects model to the random-effects model (Figs. 8 and 9).

### Adverse reactions

Borghi et al. [20] reported that two patients in their thiazide diuretic group did not complete the study because of the development of hypotension and hypokalemia; Ettinger et al. [19] reported that 41.5% of the patients in their thiazide diuretic group withdrew from the study owing to intolerance of thiazide diuretics (presence of fatigue, dizziness, and muscle symptoms); Laerum and Larsen [17] reported that two patients in their thiazide diuretic group withdrew owing to the development of hypokalemia or gout and three patients withdrew owing to the presence of mild fatigue and indigestion; Ohkawa et al. [15] mentioned that six patients in their thiazide diuretic group developed dizziness; two patients, weakness; and one patient, general malaise; and Scholz et al. reported that one patient in their thiazide diuretic group and another patient in their placebo group withdrew owing to the development of side effects. Further, 11 patients in their thiazide diuretic group complained of fatigue, nausea, and hypotension during treatment; however, the symptoms were not severe enough to cause treatment interruption [9].

### Publication bias

Egger’s test was performed to analyze the incidence of stone events in the patients with recurrent renal calculi treated with thiazide diuretics. The analysis revealed a *P* value of 0.855.

### GRADE rating

The quality of evidence for thiazide diuretics in reducing the incidence of kidney calculi was low; that in reducing the 24-h urinary calcium level among the patients with recurrent renal calculi was moderate (Table 2). Further, the quality of evidence for short-acting and long-acting thiazide diuretics in reducing the incidence of kidney calculi was low (Table 3).

**Table 2:** The evidence quality for thiazide diuretics reducing the incidence of kidney stones and 24-h urinary calcium level

Quality assessment							No of patients		Effect		Quality	Importance
No of studies	Design	Risk of bias	Inconsistency	Indirectness	Imprecision	Other considerations	UCa of thiazide diuretics	Placebo	Relative (95% CI)	Absolute		
Thiazide diuretics VS placebo in 24-h UCa												
4	Randomized trials	No serious risk of bias	No serious inconsistency	No serious indirectness	Serious^a^	None	145	171	–	SMD 18.59 lower (25.11 to 12.08 lower)	Moderate	Important
Thiazide diuretics VS placebo in incidence of new kidney stones												
8	Randomized trials	No serious risk of bias	Serious^b^	No serious indirectness	Serious^a^	None	52/286 (18.2%)	119/285 (41.8%)	–	418 fewer per 1000 (from 418 fewer to 418 fewer)	Low	Critical

*UCa* urinary calcium

^a^The sample size is not large enough and the event rate is not high enough

^b^The outcome of 2 studies is no effect

**Table 3:** The evidence quality for short-acting and long-acting thiazide diuretics reducing the incidence of kidney stones

Quality assessment							No of patients		Effect		Quality	Importance
No of studies	Design	Risk of bias	Inconsistency	Indirectness	Imprecision	Other considerations	Thiazide diuretics	Placebo in recurrent renal calculus	Relative (95% CI)	Absolute		
Subgroup—short-acting												
3	Randomized trials	No serious risk of bias	Serious	No serious indirectness	Serious^a,b^	None	27/98 (27.6%)	46/101 (45.5%)	See comment	182 fewer per 1000 (from 50 fewer to 310 fewer)	Low	Important
								48%		192 fewer per 1000 (from 53 fewer to 326 fewer)		
Subgroup—long-acting												
5	Randomized trials	No serious risk of bias	Serious^a,b^	No serious indirectness	Serious^a,b^	None	25/188 (13.3%)	73/184 (39.7%)	See comment	262 fewer per 1000 (from 179 fewer to 349 fewer)	Low	Important
								42.9%		283 fewer per 1000 (from 193 fewer to 378 fewer)		

^a^The outcome of one study is no effect

^b^The sample size is not large enough

### Decision form of recommendations

The overall strength of recommendation for the prevention of recurrent renal calculi using thiazide diuretics was not recommended (Table 4).

**Table 4:** General recommendation strength of thiazide diuretics preventing recurrent renal stones

Factor	Decision making
Quality of the evidence (The higher the quality of the evidence, the more likely a strong recommendation is warranted.)	Low
Balance of benefits versus harms and burdens (The larger the difference between the benefits and harms, the more likely a strong recommendation is warranted. The smaller the net benefit and the lower the certainty for that benefit, the more likely a conditional recommendation is warranted.)	Benefits and harms are balanced
Values and preferences (The greater the variability or uncertainty in values and preferences, the more likely a conditional recommendation is warranted.)	Major variability
Resource use (The higher the costs of an intervention, that is, the more resources consumed, the more likely a conditional recommendation is warranted.)	Moderate resource-intensive
Overall recommended strength	Low ↑?

## Discussion

Satisfactory treatment outcomes have not yet been obtained for kidney calculi, and their recurrence rate remains a concern [21–24]. Previously, multiple RCTs and reviews have reported a decreased incidence of recurrent kidney calculi with diet control (e.g., high fluid intake and calcium intake limitation) and thiazide diuretic, alkali citrate, and allopurinol administration. Herein, the overall efficacy of thiazide diuretics in reducing the incidence of recurrent renal calculi was limited when compared to the placebo and untreated groups and the pooled RD with 95% CI was − 0.23 (− 0.30 to − 0.16). The quality of evidence was low owing to the insufficient samples and the inconsistent results among the studies when the GRADE criteria were used to assess the incidence of stone events. This result considerably decreased our expectation of the efficacy of thiazide diuretics in reducing the incidence of recurrent kidney calculi.

We reviewed the clinical guidelines and found that the 2016 updated edition of the Canadian Urological Association guidelines considered thiazide diuretics as a highly recommended drug for preventing recurrent kidney calculi (level of evidence: 1–3 and grade A–B recommendation, based on the Oxford levels of evidence and grades of recommendation). However, the grade according to the American College of Physicians guidelines was weak (grade: weak recommendation, moderate-quality evidence).

In addition to concerns regarding stone events, we also observed a decrease in the serum potassium level and an increase in the uric acid level in some thiazide diuretic groups among the eight studies. Further, 3.7–20% of the patients in the thiazide diuretic groups withdrew from the trials owing to the development of adverse reactions (e.g., hypokalemia, elevated uric acid levels, and abnormal blood glucose and cholesterol levels). Clinically, the main adverse reactions of thiazide diuretics are as follows: (1) water and electrolyte disturbance, such as hypokalemia and hyponatremia; (2) cardiovascular problems, such as blood volume insufficiency and orthostatic hypotension; (3) gastrointestinal reactions, such as anorexia, nausea, gastric irritation, and constipation; (4) central nervous system problems, such as dizziness, paresthesia, and headache; and (5) abnormalities in related metabolic indicators, such as hyperglycemia and elevated total cholesterol levels [25–27]. Makam et al. [28] showed that 14.3% of thiazide users and 6.0% of non-users had adverse reactions (serum sodium level of < 135 mmol/L; serum potassium level of < 3.5 mmol/L; and estimated glomerular filtration rate reduction by > 25% compared with that at baseline) (*P *< 0.05). In the ALLHAT trial, the incidence of newly diagnosed diabetes was 17.1% in patients with metabolic syndrome after using chlorthalidone for 4 years; that in patients without metabolic syndrome was 7.7% (*P *< 0.05) [29].

However, the patients’ compliance was poor. Dauw et al. [30] found that when patients with kidney calculi were treated with a single prophylactic drug, the proportion of patients in the thiazide diuretic groups who followed the doctor’s advice was only 42.5%. Even in the presence of cardiovascular diseases (e.g., hypertension), patients in the thiazide diuretic groups were equally less docile (42%) [31].

Taken together, thiazide diuretics yielded several adverse reactions, and the patients’ compliance was low. Based on the decision table formed by the recommendation in the WHO Handbook for Guideline Development and combined with the degree of benefits for patients with kidney calculi, patients’ compliance, adverse reactions caused by long-term medication, and economic burden, thiazides are not recommended for use in preventing recurrent kidney calculi.

Our study also has some limitations: (1) although we searched each database using keywords, not all relevant documents were included, such as unpublished literature; (2) most of the studies included have been conducted several years ago; (3) the kidney calculi investigated in this study were all calcium stones. Whether thiazide diuretics have any effect on other types of kidney calculi remains unclear; thus, the results of this meta-analysis should be interpreted with caution; (4) some of the included studies assessed experimental groups and control groups with other measures as diet, fluid therapy or potassium chloride, which may be a source of heterogeneity; and (5) we only described the adverse reactions qualitatively and didn’t conduct meta-analysis of the safety of thiazide diuretics for recurrent kidney calculi.

## Conclusions

Long-term use of thiazide diuretics can reduce the incidence of recurrent renal calculi and 24-h urinary calcium level. However, the benefits are insufficient, and the quality of evidence is low. Considering the adverse effects, patients’ preferences, and economic burden of long-term medication, we do not recommend the use of thiazide diuretics to prevent recurrent kidney calculi.

## Supplementary information

**Additional file 1: Table S1.** Search strategy of PubMed.

## Data Availability

Not applicable.

## Abbreviations

RCT: Randomized controlled trial
RR: Relative risk
RD: Risk difference
SMD: Standardized mean difference
CI: Confidence interval
