# Supplementary material for: Use of thiazide diuretics for the prevention of recurrent kidney calculi: a systematic review and meta-analysis
Source: J Transl Med. 2020 Feb 28;18:106. doi: 10.1186/s12967-020-02270-7 (PMC7048029; doi:10.1186/s12967-020-02270-7)
Supplement: Supplementary file 1 — Additional file 1: Table S1. Search strategy of PubMed. [file 12967_2020_2270_MOESM1_ESM.doc]

**Search strategy of PubMed**

| Search | Query | Items found |
| --- | --- | --- |
| #1 | Search "Sodium Chloride Symporter Inhibitors"[Mesh] Sort by: [pubsolr12] | 2975 |
| #2 | Search (((((((("Sodium Chloride Symporter Inhibitors"[Mesh]) OR Sodium Chloride Cotransporter Inhibitors[Title/Abstract]) OR Thiazide Sensitive NaCl Cotransporter Inhibitors[Title/Abstract]) OR Diuretics, Thiazide[Title/Abstract]) OR Thiazide Diuretics[Title/Abstract]) OR Benzothiadiazine Diuretics[Title/Abstract]) OR Diuretics, Benzothiadiazine[Title/Abstract]) OR Potassium Depleting Diuretics[Title/Abstract]) OR Diuretics, Potassium Depleting[Title/Abstract] Sort by: [pubsolr12] | 4300 |
| #3 | Search "Kidney Calculi"[Mesh] Sort by: [pubsolr12] | 18578 |
| #4 | Search (((((((((((("Kidney Calculi"[Mesh]) OR Calculi, Kidney[Title/Abstract]) OR Calculus, Kidney[Title/Abstract]) OR Kidney Calculus[Title/Abstract]) OR Nephrolith[Title/Abstract]) OR Renal Calculus[Title/Abstract]) OR Kidney Stones[Title/Abstract]) OR Kidney Stone[Title/Abstract]) OR Stone, Kidney[Title/Abstract]) OR Stones, Kidney[Title/Abstract]) OR Renal Calculi[Title/Abstract]) OR Calculi, Renal[Title/Abstract]) OR Calculus, Renal[Title/Abstract] Sort by: [pubsolr12] | 22210 |
| #5 | Search (((((((((((((("Kidney Calculi"[Mesh]) OR Calculi, Kidney[Title/Abstract]) OR Calculus, Kidney[Title/Abstract]) OR Kidney Calculus[Title/Abstract]) OR Nephrolith[Title/Abstract]) OR Renal Calculus[Title/Abstract]) OR Kidney Stones[Title/Abstract]) OR Kidney Stone[Title/Abstract]) OR Stone, Kidney[Title/Abstract]) OR Stones, Kidney[Title/Abstract]) OR Renal Calculi[Title/Abstract]) OR Calculi, Renal[Title/Abstract]) OR Calculus, Renal[Title/Abstract])) AND ((((((((("Sodium Chloride Symporter Inhibitors"[Mesh]) OR Sodium Chloride Cotransporter Inhibitors[Title/Abstract]) OR Thiazide Sensitive NaCl Cotransporter Inhibitors[Title/Abstract]) OR Diuretics, Thiazide[Title/Abstract]) OR Thiazide Diuretics[Title/Abstract]) OR Benzothiadiazine Diuretics[Title/Abstract]) OR Diuretics, Benzothiadiazine[Title/Abstract]) OR Potassium Depleting Diuretics[Title/Abstract]) OR Diuretics, Potassium Depleting[Title/Abstract]) Sort by: [pubsolr12] | 152 |
| #6 | |  | Search (((Randomized Controlled Trial[Publication Type]) OR Randomized Controlled Trial[Title/Abstract]) OR Randomized Controlled Trial[Filter]) OR Random*[Title/Abstract] Sort by: Best Match | | --- | --- | | 1215778 |
| #7 | Search (((((Randomized Controlled Trial[Publication Type]) OR Randomized Controlled Trial[Title/Abstract]) OR Randomized Controlled Trial[Filter]) OR Random*[Title/Abstract])) AND ((((((((((((((("Kidney Calculi"[Mesh]) OR Calculi, Kidney[Title/Abstract]) OR Calculus, Kidney[Title/Abstract]) OR Kidney Calculus[Title/Abstract]) OR Nephrolith[Title/Abstract]) OR Renal Calculus[Title/Abstract]) OR Kidney Stones[Title/Abstract]) OR Kidney Stone[Title/Abstract]) OR Stone, Kidney[Title/Abstract]) OR Stones, Kidney[Title/Abstract]) OR Renal Calculi[Title/Abstract]) OR Calculi, Renal[Title/Abstract]) OR Calculus, Renal[Title/Abstract])) AND ((((((((("Sodium Chloride Symporter Inhibitors"[Mesh]) OR Sodium Chloride Cotransporter Inhibitors[Title/Abstract]) OR Thiazide Sensitive NaCl Cotransporter Inhibitors[Title/Abstract]) OR Diuretics, Thiazide[Title/Abstract]) OR Thiazide Diuretics[Title/Abstract]) OR Benzothiadiazine Diuretics[Title/Abstract]) OR Diuretics, Benzothiadiazine[Title/Abstract]) OR Potassium Depleting Diuretics[Title/Abstract]) OR Diuretics, Potassium Depleting[Title/Abstract])) Sort by: Best Match | 18 |
